# Post-transplant Replication of Torque Teno Virus in Granulocytes

**DOI:** 10.3389/fmicb.2018.02956

**Published:** 2018-11-29

**Authors:** Karin Kosulin, Silvia Kernbichler, Herbert Pichler, Anita Lawitschka, René Geyeregger, Volker Witt, Thomas Lion

**Affiliations:** ^1^Children’s Cancer Research Institute, Vienna, Austria; ^2^St. Anna Children’s Hospital, Vienna, Austria; ^3^Department of Pediatrics, Medical University of Vienna, Vienna, Austria

**Keywords:** Torque Teno virus, allogeneic stem cell transplantation, immunosuppression, pediatric patients, granulocytes

## Abstract

Torque Teno virus (TTV) in humans is characterized by ubiquitous occurrence in peripheral blood (PB), without any related disease described to date. Several studies reported a significant increase of TTV plasma DNA levels in allogeneic transplant recipients, and suggested a correlation of elevated virus titers with immunosuppression and transplant-related complications. However, the site of viral replication in this setting has remained unclear. We have studied TTV in serial plasma specimens derived from 43 pediatric allogeneic hematopoietic stem cell transplantation (HSCT) recipients by RQ-PCR, and found increasing TTV-DNA levels in all patients post-transplant, with a peak around day +100 and maximum virus copy numbers reaching 4 × 10E9/ml. To assess whether the virus replicates in PB-cells, leukocyte subsets including granulocytes, monocytes, NK-cells, T- and B-lymphocytes were serially isolated by flow-sorting for TTV analysis in 19 patients. The virus was undetectable in most cell types, but was identified in granulocytes in all instances, revealing a median DNA copy number increase of 1.8 logs between days +30–100 post-transplant. Our data therefore provide evidence for TTV replication in granulocytes in this setting. In a control cohort of immunocompetent children and in HSCT recipients before day +30, TTV positivity in granulocytes was less common (33%), and the copy numbers were considerably lower. However, rising TTV replication about 2 weeks after granulocyte engraftment (>500 cells/μl) was observed suggesting that granulocyte recovery might be required for TTV expansion in severely immunosuppressed transplant recipients.

## Introduction

Torque Teno Virus (TTV) was first discovered in 1997 in a patient with hepatitis of unknown etiology (Nishizawa et al., 1997), and was assigned to the family of Anelloviridae (Maggi and Bendinelli, 2009; Spandole et al., 2015). The prevalence of TTV in the general population was described to reach up to 90%, with detectability of the virus in peripheral blood (PB), stool, saliva, and pharyngeal mucus, suggesting several possible routes of transmission and a high probability of exposure (Okamoto et al., 1998; Deng et al., 2000; Maggi et al., 2003; Chung et al., 2007; Okamoto, 2009). Although TTV apparently establishes long-lasting persistent infections and circulates in PB in most individuals, no convincing evidence for its potential pathogenicity in humans has been provided to date (Bendinelli et al., 2001). The common occurrence of TTV in samples derived from whole PB and plasma raised the possibility that hematopoietic cells might serve as a reservoir for the virus, and a few studies indeed reported detection of TTV in B and T lymphocytes, monocytes and granulocytes, but neither in platelets nor in erythrocytes (Maggi et al., 2001; Takahashi et al., 2002). Nevertheless, the sites of virus persistence and replication have remained uncertain due to the lack of conclusive evidence. The detection of TTV has not been clearly associated with any disease in humans, but a correlation between the virus levels in plasma and the degree of immunosuppression has been discussed in the context of decreased graft rejection rates observed in solid organ transplant recipients with high TTV titers (De Vlaminck et al., 2013; Schiemann et al., 2017). The correlation with immune reconstitution in the transplant setting is supported by observations of rising and subsequently decreasing TTV plasma levels in patients after lung or liver transplantation (Burra et al., 2008; Maggi et al., 2010; Gorzer et al., 2015) and hematopoietic stem cell transplantation (HSCT) (Focosi et al., 2010; Masouridi-Levrat et al., 2016). Recent studies therefore discussed the possibility of exploiting TTV DNA levels in plasma or whole PB as a biomarker for the risk of severe immune reactions and immune recovery after HSCT (Albert et al., 2017; Gilles et al., 2017; Wohlfarth et al., 2018). Although the potential clinical benefit of performing quantitative TTV monitoring in transplant recipients has been stressed in these studies, current evidence supporting this concept is rather limited. Additional studies would be required to corroborate previous observations and to provide more detailed insights pertaining to the mechanisms of TTV increase after engraftment to elucidate the potential role of the virus in the transplant setting.

In the present study, consecutive plasma samples derived from 43 pediatric allogeneic HSCT recipients were monitored for TTV levels over a time period of more than 1 year post-transplant. The potential site(s) of TTV replication in individual blood cell types were assessed by investigating the virus copy numbers in various leukocyte subsets isolated by flow-sorting. The study performed in pediatric HSCT recipients provides evidence for TTV replication in granulocytes which culminated around 100 days post-transplant. The onset and kinetics of virus replication appeared to correlate with the level of immunosuppression and the time course of granulocyte engraftment.

## Patients and Methods

### Patients

Archived plasma, stool, PB, and bone marrow (BM) specimens derived from pediatric allogeneic stem cell transplant recipients treated at the St. Anna Children’s Hospital, Vienna, Austria, were used for the study. The specimens had been collected as part of routine diagnostic virus screening at consecutive time points starting before conditioning followed by collection at 7-day intervals until day +100, thereafter at 2 to 3 week intervals until day +360, and at less regular intervals after the first year post-transplant. Written informed consent was obtained from each of the 43 patients and/or the parents. The patient characteristics are listed in Table 1. A control cohort including 60 immunocompetent patients treated at St. Anna Children’s Hospital was studied in parallel. From these patients, residual anonymized PB samples remaining from routine laboratory analyses had been collected. Approval for analysis of TTV in the collected samples was obtained from the Ethics Committee (Medical University Vienna, Austria) EK Nr: 2069/2016. In addition, expired platelet concentrates from 14 donors were kindly provided for TTV testing by the Center for Blood Donations of the Austrian Red Cross.

**Table 1:** Characteristics of the HSCT recipients investigated and TTV copies/ml plasma.

Pat	Donor	Underlying disease	ATG	Cond.	^a^CD3^+^	^a^NK	GvHD grade	TTV^b^ Before HSCT 0	TTV^b^ Post-HSCT^c^	Log difference
1	UD	Malignant	Yes	MA	19	12	2	0	8.87E+06	6.98
2	MSD	Malignant	Yes	MA	27	20	1	0	5.24E+07	7.72
3	MSD	Malignant	Yes	MA	20	13	2	0	4.75E+05	5.68
4	MSD	Malignant	Yes	RIC	20	14	0	0	2.86E+05	5.46
5	UD	SCID	Yes	RIC	62	20	1	0	7.38E+03	3.87
6	UD	Malignant	No	MA	13	13	0	0	3.02E+07	7.48
7	MMFD	SCID	No	RIC	36	12	0	0	4.40E+08	8.64
8	MSD	Malignant	Yes	MA	11	11	2	0	4.07E+09	9.61
9	UD	Malignant	Yes	MA	27	27	0	0	2.59E+08	8.41
10	MMFD	Malignant	Yes	RIC	15	21	4	1.16E+04	5.31E+08	4.66
11	UD	Malignant	No	MA	13	17	1	3.81E+05	1.22E+09	3.51
12	UD	Malignant	No	MA	13	43	2	1.37E+04	2.43E+06	2.25
13	UD	Malignant	Yes	RIC	20	13	0	9.85E+02	1.01E+09	6.01
14	UD	Malignant	Yes	MA	26	16	2	4.24E+04	1.07E+08	3.40
15	UD	SCID	Yes	RIC	14	14	2	1.50E+03	1.01E+09	5.83
16	MMFD	Malignant	No	RIC	27	20	0	7.01E+04	1.07E+08	3.84
17	UD	Malignant	No	MA	13	9	1	6.42E+03	1.01E+09	4.82
18	UD	Malignant	Yes	RIC	28	7	1	4.31E+03	4.89E+08	5.06
19	UD	SCID	Yes	RIC	13	13	1	6.45E+06	4.27E+08	1.67
20	UD	SCID	No	RIC	30	16	0	1.91E+07	4.97E+08	0.78
21	UD	Malignant	Yes	MA	26	26	0	5.34E+03	6.88E+07	4.11
22	MSD	Malignant	Yes	RIC	18	18	0	2.13E+07	2.13E+07	0.00
23	UD	Malignant	Yes	RIC	69	10	0	4.65E+04	3.06E+07	2.82
24	MMFD	Malignant	Yes	RIC	13	13	4	1.30E+06	2.66E+09	3.31
25	UD	SCID	Yes	RIC	8	13	3	8.10E+05	2.31E+07	1.45
26	MSD	Malignant	Yes	RIC	14	14	0	2.34E+04	8.74E+07	3.57
27	UD	Malignant	Yes	RIC	13	13	0	4.56E+04	4.15E+08	3.96
28	MMFD	Fanconi A.	Yes	RIC	8	13	0	1.27E+04	2.05E+07	3.21
29	MMFD	Malignant	Yes	MA	10	13	3	2.46E+03	5.20E+08	5.32
30	UD	Malignant	Yes	RIC	22	14	0	1.61E+05	2.32E+08	3.16
31	UD	Malignant	Yes	RIC	34	27	0	2.31E+05	2.31E+05	0.00
32	UD	Malignant	No	MA	14	14	0	4.67E+04	7.41E+07	3.20
33	UD	Malignant	Yes	MA	25	18	2	4.28E+05	1.24E+09	3.46
34	MSD	Malignant	Yes	MA	19	12	3	6.66E+05	1.25E+06	0.27
35	MSD	SCID	Yes	RIC	14	14	4	7.55E+08	1.85E+09	0.39
36	UD	Malignant	Yes	RIC	10	10	0	3.08E+03	l.72E+05	1.75
37	UD	Malignant	No	MA	n.a.	9	3	8.24E+05	8.30E+08	3.00
38	MSD	Malignant	Yes	MA	63	33	0	1.36E+04	l.28E+08	3.97
39	UD	Malignant	Yes	MA	25	18	1	3.39E+03	8.33E+07	4.39
40	UD	Malignant	No	MA	13	13	1	6.78E+04	5.21E+08	3.89
41	UD	Malignant	Yes	MA	25	25	3	9.92E+06	2.14E+08	1.33
42	MSD	Malignant	No	MA	13	13	3	2.44E+04	1.06E+07	2.64
43	UD	Malignant	Yes	MA	28	28	4	5.48E+05	6.09E+07	2.05

*^*a*^Engraftment at the indicated days after HSCT; ^*b*^DNA copies/ml plasma; ^*c*^highest value within 100 days post-transplant; UD, unrelated donor; MSD, matched sibling donor; MMFD, mismatched family donor; A., anemia; SCID, severe combined immunodeficiency; ATG, anti-thymocyte globulin; Cond., conditioning; RIC, reduced intensity conditioning; MA, myeloablative conditioning; n.a., not available*.

### Specific Cell Separation by Centrifugation and Flow Cytometry

Individual leukocyte subsets were isolated from PB or BM via staining for specific cell surface antigens including CD4^+^, CD8^+^, CD19^+^, CD56^+^, CD33^+^, and CD15^+^ as part of routine diagnostics in HSCT recipients at the St. Anna Children’s Hospital, Vienna, Austria. Cell sorting was performed on a FACSAria instrument (BD Biosciences) using the FACSDiVa software, as described previously (Fritsch et al., 1999). The purity of individual leukocyte fractions was >98%. Generally, 4,000 cells from each leukocyte subset were isolated for subsequent analysis. For DNA isolation from plasma, PB samples were centrifuged at 100 *g* for 10 min, and the cell pellets were discarded. Erythrocytes derived from PB specimens of patients from the control group were obtained upon cell sedimentation. The platelet concentrates were centrifuged at 1,600 *g* for 30 min and resuspended in PBS for DNA isolation, as described below. Erythrocyte and platelet numbers were determined by a Sysmex Kx21 Hematology Analyzer (Sysmex).

### Isolation of Viral DNA and RNA

The QIAamp DNA Mini Kit (Qiagen) was employed for the isolation of DNA from plasma, PB and individual cell subsets. Extraction of DNA from stool specimens was performed by the QIAamp DNA Stool Mini Kit (Qiagen) according to the manufacturer’s recommendations, and viral RNA was isolated with the RNeasy Kit plus (Qiagen), with subsequent DNA digestion with DNase Q1 (Promega). For further real-time quantitative (RQ)-PCR analyses, the RNA was transcribed into cDNA using the MLV reverse transcriptase (Promega).

### TTV DNA Detection by RQ-PCR

RQ-PCR targeting the UTR region of the TTV genome was used for virus detection. The PCR assay previously described by Maggi et al. (2003) was modified by adding an additional reverse primer (5′-GGTCCGGCCAGTCC-3) to cover a greater spectrum of TTV strains. Moreover, a Taqman probe labeled with a fluorescence dye and a quencher, but without further modifications (5′FAM-TCAAGGGGCAATTCGGGCT-TAMRA3′), was employed. The detection limit, linearity and reproducibility of the assay were determined by using quantified plasmid preparations containing the UTR region of TTV (Maggi et al., 2001) (kindly provided by F. Maggi, University of Pisa, Italy) and appropriate standard curves. The consistently achievable detection limit of the assay was 10 TTV DNA copies per PCR reaction. To determine the virus copy numbers per cell, a human single-copy housekeeping gene (beta2-microglobulin) was co-amplified by RQ-PCR in parallel with the virus-specific assay, as described previously (Watzinger et al., 2004). All RQ-PCR analyses were performed on the ABI Prism Fast 7500 instrument (Thermo Fisher Scientific).

### TTV RNA Detection by RQ-PCR

RQ-PCR assays exploiting SYBR green were used to analyze the TTV RNA expression. The Maxima SYBR green Master Mix (Thermo Fisher Scientific) with the following oligonucleotides was used: TTV1 forI 5′-GACACAGAACTCACAGCCC-3′, TTV1 forII 5′-GACACTGACGTGACAGCCG-3′, TTV1 rev 5′-GTTAGTGGTGAGCCGAACG-3′. The PCR was performed with 10 pmol of each oligonucleotide targeting the VP1 region of the TTV genotype 1, at an annealing temperature of 60°C, according to the protocol recommended for the Maxima SYBR green Master Mix.

### Statistics

Spearman correlation was employed for analysis of the correlation coefficient between TTV levels in plasma and lymphocyte as well as granulocyte counts on days +30, +60, and +100 post-transplant. The calculations were performed by employing the GraphPad Prism 5 software.

## Results

### Expansion of TTV in Plasma and Stool After HSCT

**FIGURE 1:**
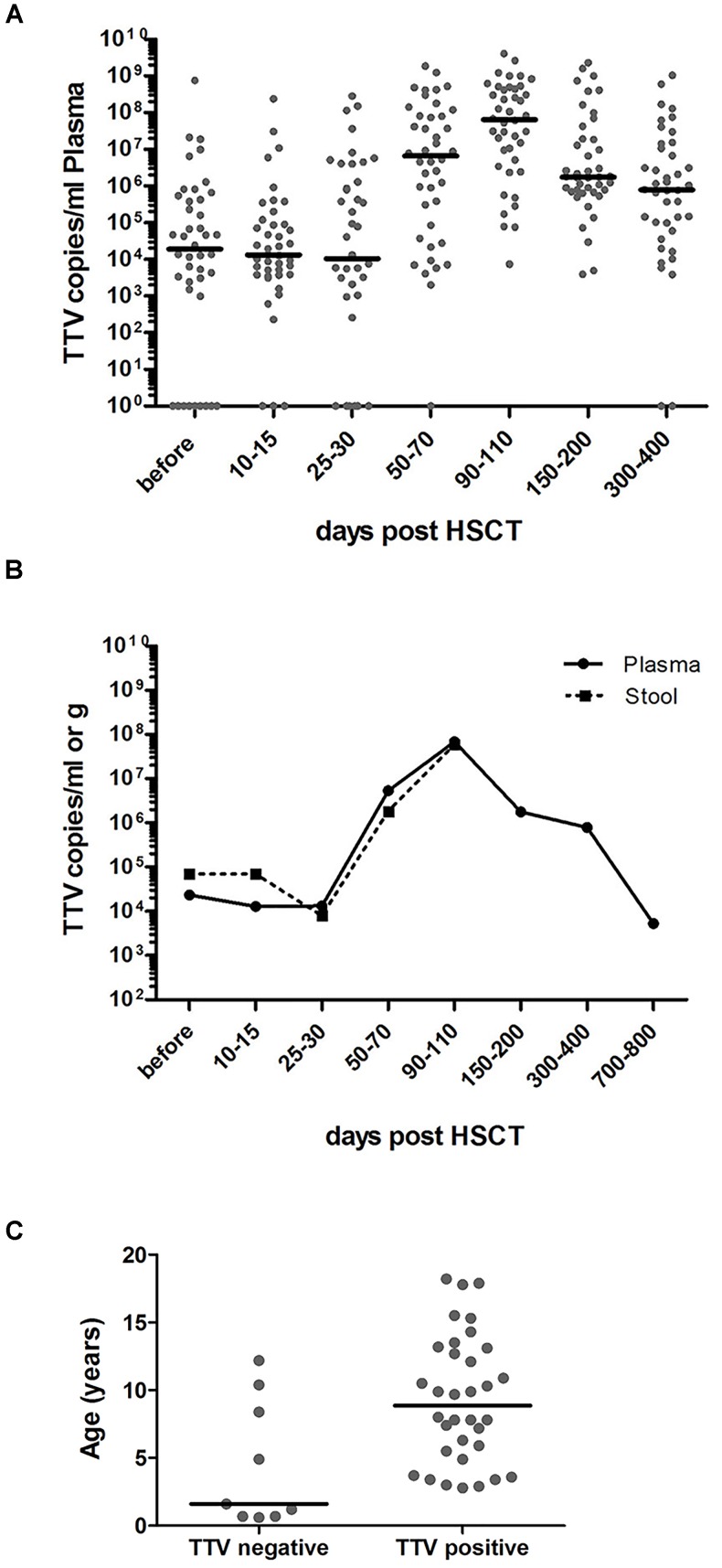
RQ-PCR monitoring of TTV copies in plasma and stool before and after HSCT. RQ-PCR analysis detecting TTV was exploited to analyze the virus load. **(A)** The dot plot depicts the numbers of TTV copies/ml plasma in individual pediatric patients (*n* = 43) during the indicated time periods before and after HSCT. The horizontal bars represent the respective median values. **(B)** The curves reflect the median values of TTV copies per ml plasma (*n* = 43; solid line) or per gram stool (*n* = 21; dashed line) during the indicated observation period. **(C)** The age of patients at the time of transplantation is shown in correlation with TTV negativity and positivity in plasma pre-transplant (*t*-test; *p* < 0.01).

Elevated TTV titers were previously described primarily in adult solid organ transplant (SOT) recipients and in a small number of recent studies in patients undergoing HSCT (Burra et al., 2008; Focosi et al., 2010; Gorzer et al., 2015; Wohlfarth et al., 2018). In the present study, 34 of the 43 pediatric HSCT patients investigated (79%) had the virus detectable in plasma already before transplantation, and all patients tested positive for TTV DNA by day +50 post-transplant, as revealed by RQ-PCR analysis. The TTV DNA copy numbers before HSCT and the highest copy numbers detected in plasma until day +100 post-transplant are shown in Table 1, along with pertinent patient characteristics including the conditioning regimen, *in vivo* T cell depletion (involving primarily ATG), day of engraftment, and grade of GvHD, if present. In TTV positive patients, the median virus load detected before transplantation and until day +30 post-transplant was 1 × 10E4 DNA copies per ml plasma, with rapidly rising levels after day +30. The peak was generally reached around day +100, with a median of 7 × 10E7 (range: 7 × 10E3 – 4 × 10E9) virus copies per ml plasma (Figure 1A). The TTV DNA copy numbers observed within defined sequential time periods after transplantation were highly variable, occasionally revealing even switches between negativity and positivity, but the patients analyzed invariably tested positive for TTV between days +90 and +200 post-transplant (Figure 1A). After day +100, gradually decreasing virus loads were observed, and the median time for returning to pre-transplant TTV levels was 2 years (Figure 1B). Monitoring of TTV levels in serial stool samples until day +100 was performed in 21 of the 43 patients investigated, and revealed median virus levels and replication kinetics very similar to the findings in plasma at corresponding time points (Figure 1B). A significant age dependency of TTV positivity in plasma before HSCT has been observed revealing a median age of 1.6 years (range: 0.6–12 years) in patients who tested TTV negative pre-transplant, and 9 years (range: 3–18 years) in patients who were TTV positive in plasma already before HSCT (Figure 1C, *p* < 0.01).

### Delayed Post-transplant Onset of TTV Replication

**FIGURE 2:**
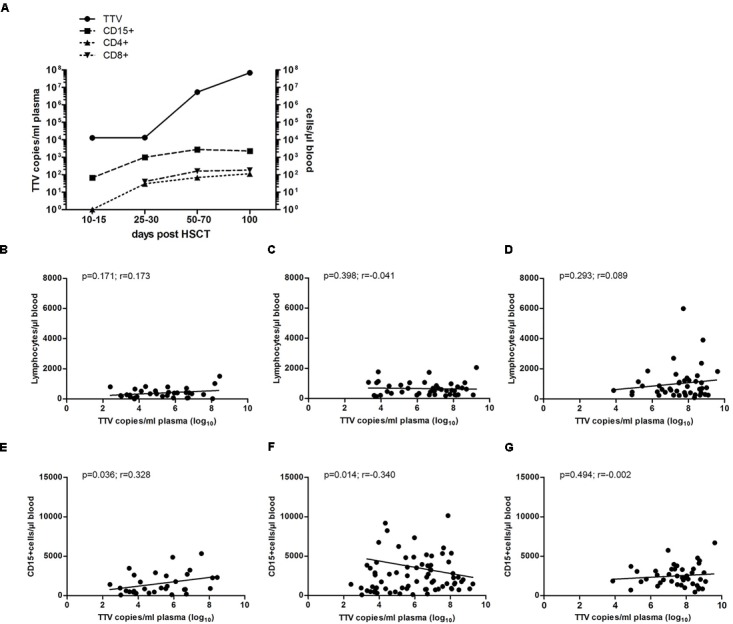
Correlation of TTV plasma levels with granulocyte and lymphocyte numbers. Median TTV DNA copy numbers per ml plasma were analyzed by RQ-PCR. **(A)** The kinetics of TTV load (solid line) representative of all 43 patients investigated are shown in relation to the median cell numbers of granulocytes, CD4^+^ and CD8^+^ cells (dashed lines) at the indicated time windows post-HSCT. Spearman correlation of TTV levels and total lymphocyte counts at days **(B)** +30, **(C)** +60, and **(D)** +100 post-HSCT and **(E–G)** granulocyte counts (CD15^+^ cells) at days +30, +60, and +100 post-HSCT, respectively, is shown for the entire cohort of 43 patients. The *p*-values and correlation coefficients (r) are indicated.

Based on different studies performed in the adult transplant setting, which indicated the possibility of exploiting the TTV kinetics as a marker for the immune status in HSCT recipients (Focosi et al., 2010; Albert et al., 2017; Gilles et al., 2017), we have addressed the potential clinical use of TTV monitoring in a cohort of pediatric patients undergoing allogeneic HSCT. During the first month post-transplant, the median TTV DNA copy numbers in plasma remained more or less constant, while the median numbers of generally donor-derived CD4^+^, CD8^+^ cells and granulocytes increased by more than one log within this time interval. Subsequently, after day +30 post-transplant, following T cell and neutrophil engraftment, rapidly rising TTV DNA copy numbers were observed, with peak levels around day +100 (Figure 2A). During this time period, the median T cell and granulocyte numbers had already reached plateau levels above 250/μl and 2500/μl blood, respectively. The TTV plasma levels therefore showed no significant correlation with lymphocyte counts between days +30 and +100 post-HSCT (Figures 2B–D), and the lack of correlation also applied to the T cell subsets CD4^+^ and CD8^+^ (data not shown). However, the TTV plasma levels correlated significantly with the granulocyte counts on days +30 and +60 (*p* < 0.05; Figures 2E,F), but not on day +100 post-HSCT (Figure 2G). There was no correlation between TTV plasma levels at these time points and the absence or presence of GvHD at any level (data not shown).

### Assessment of the TTV Replication Sites and Expansion Kinetics in Transplant Recipients

**FIGURE 3:**
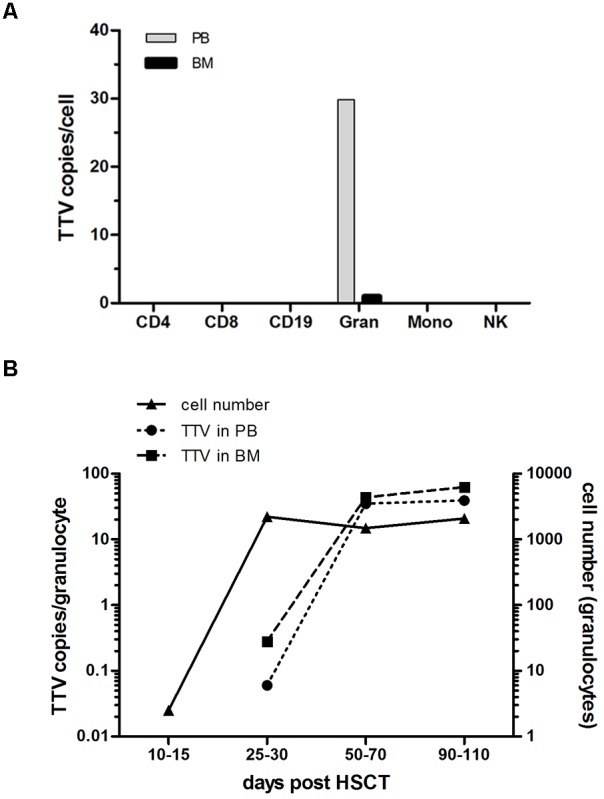
TTV detection in leukocyte subsets and virus replication kinetics after transplantation. Leukocyte subsets including CD4^+^, CD8^+^, CD19^+^, CD15^+^ (granulocytes: Gran), CD33^+^ (monocytes: Mono) and CD56^+^ (natural killer cells: NK) were isolated by flow sorting from peripheral blood (PB) and bone marrow (BM) of 19 pediatric HSCT recipients. Individual cell subsets were tested for the presence and quantity of virus copies by a TTV-specific RQ-PCR assay. **(A)** The median of the peak TTV copy numbers per cell determined in the patients investigated is shown for the different leukocytes subsets. **(B)** The TTV replication kinetics in granulocytes isolated from PB and BM of the 19 patients investigated is shown by the median virus copy numbers per cell at the indicated time points post-HSCT. The *y*-axis on the right side indicates the granulocyte numbers per μl PB.

To address the possibility that post-transplant replication of TTV occurs in specific nucleated blood cells, different leukocyte subsets were isolated by flow-sorting from PB or BM of 19 pediatric allogeneic HSCT recipients displaying a donor chimerism exceeding 98% beyond day +50 post-transplant. Testing for the presence of TTV DNA in individual leukocyte subsets was performed by RQ-PCR analysis of DNA derived from 4,000 cells per subset. While T cells including the CD4^+^ and CD8^+^ subpopulations, B cells and NK cells did not show any evidence for the virus, granulocytes (CD15^+^) isolated either from PB or BM tested invariably positive in post-transplant samples of all patients analyzed (Figure 3A). Monocytes derived from BM specimens revealed TTV-positive results in three of the 19 patients analyzed, but the median copy numbers were too low (<0.6 copies/cell) to generate a visible signal in the panel displayed (Figure 3A). For the assessment of TTV positivity in individual leukocyte subsets, it was necessary to consider that the purity of flow-sorted cell fractions was generally >98%, thus enabling low-level contamination with other leukocyte subsets. Since contamination with TTV-positive granulocytes could have resulted in false-positive test results for individual leukocyte fractions, low-level positivity below 2% of the TTV DNA copy numbers detected in leukocyte subsets other than granulocytes was regarded as a negative result. However, the three patients who tested TTV-positive in the monocyte fractions showed virus copy numbers above the indicated threshold, suggesting the presence of true positive test results. The kinetics of increasing TTV replication in granulocytes during the first 100 days after transplantation revealed median virus copy numbers ranging from 0.1 to 40 per cell in PB and 1 to 63 per cell in BM (Figure 3B). The replication of the virus in granulocytes is documented by a roughly two-log increase of median virus copy numbers per cell between days +30 and +100 after allogeneic HSCT (Figure 3B). Before day +30 post-transplant, TTV tested either negative or only low-positive in granulocytes, although the cell numbers of this leukocyte subset expanded rapidly above 500 granulocytes per μl PB already between 2 and 4 weeks after transplantation in 17 of the 19 patients analyzed (Figure 3B). In addition expression of viral RNA was assessed in flow-sorted granulocytes derived from five HSCT-recipients between 40 to 100 days post-transplant. Expression of TTV RNA was detected in all instances. Control experiments confirmed the absence of TTV DNA in the RNA samples analyzed (data not shown).

### Persistence of TTV in Plasma and Blood Cells

**FIGURE 4:**
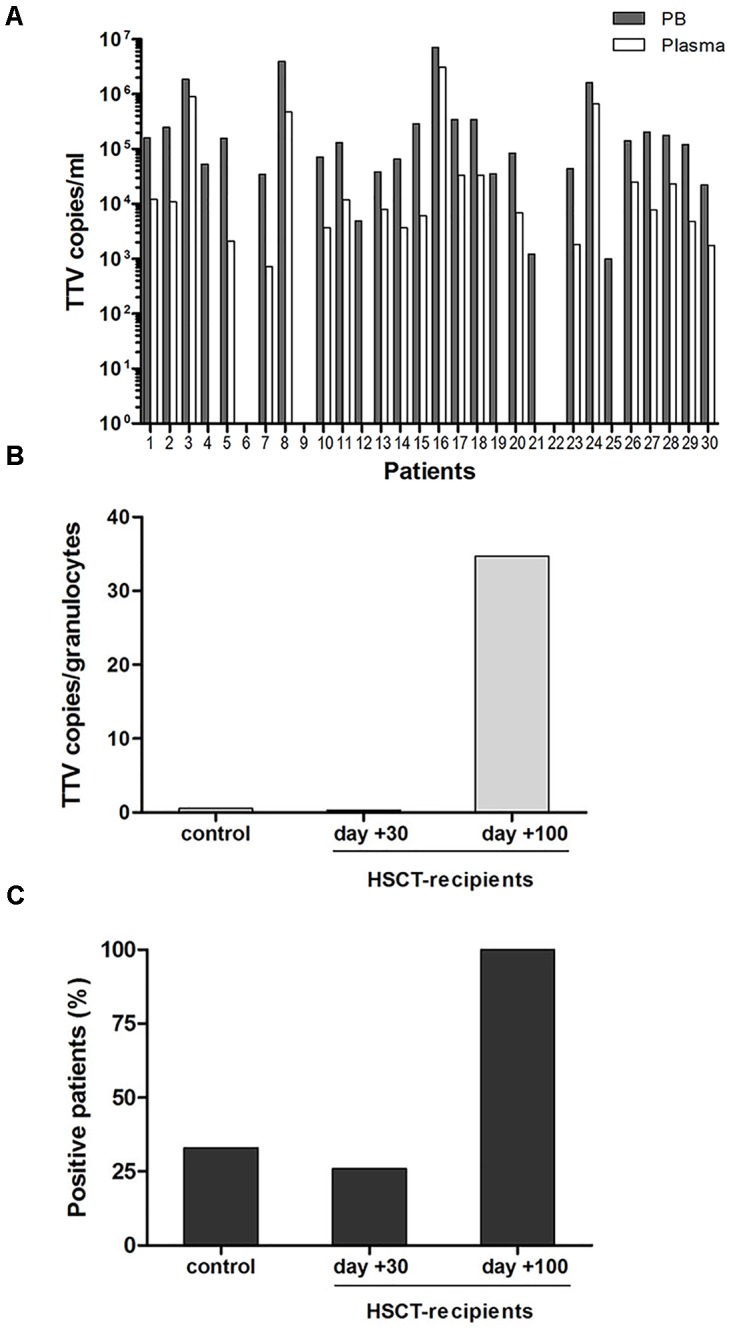
TTV in peripheral blood and granulocytes in the pediatric control cohort. **(A)** Copy numbers of TTV in peripheral blood and plasma of 30 non-oncological control patients. **(B+C)** Comparison between the control group and the HSCT recipients until days +30 and +100. **(B)** TTV copies per granulocyte (CD15^+^ cells), **(C)** patient numbers displaying TTV positive granulocytes.

To assess whether the observed TTV replication in granulocytes of pediatric HSCT recipients might correlate with transplant-independent persistence of the virus in PB, targeted testing was performed in a control cohort of 30 hospitalized children with non-malignant diseases who were no candidates for allogeneic stem cell transplantation. Testing of whole PB and plasma samples for TTV sequences by RQ-PCR revealed presence of the virus in 27/30 (90%) of the PB specimens and in 22/30 (73%) of the plasma specimens analyzed. In all instances, the virus loads detected in whole PB samples were considerably higher than in plasma samples of the same patients, suggesting common TTV association with cellular elements (Figure 4A). The median TTV DNA copy numbers in PB were 1.0E+05/ml (range: 0.0 to 7.2E+06 copies/ml), and 5.4E+03/ml (range: 0.0 to 3.1E+06 copies/ml) in plasma. In 5/30 patients who tested positive for TTV only in whole PB but not in plasma, the virus copy numbers were rather low, ranging between 1E+3 and 5E+4 per ml (Figure 4A). In patients with TTV positivity both in whole PB and plasma, the median difference in virus copy numbers was >1 log_10_ (range: 0.31–1.87 log_10_). To assess the presence of TTV in various leukocyte subsets, T and B lymphocytes (CD4^+^, CD8^+^, and CD19^+^), NK-cells, monocytes and granulocytes were isolated from PB of all 30 patients by flow-sorting, and 4,000 cells of each subset were used for DNA extraction and RQ-PCR analysis. Based on the observation of TTV positivity in granulocytes and monocytes of HSCT recipients, an additional 100,000 cells of these leukocyte subsets and T cells were collected to increase the sensitivity of virus detection. Of the cell types analyzed, only granulocytes tested positive for TTV, and the virus was detected in DNA samples derived from two additional patients in whom TTV was undetectable in DNA isolated from 4,000 cells. However, only 10/30 (33%) patients from the control cohort had TTV detectable in granulocytes, and the virus copy numbers were lower than in transplant recipients (median: 0.5 TTV copies per cell; range: 0.01 to 8 TTV copies per cell). In line with this observation, 5 of the 19 transplant recipients investigated (26%) were positive for TTV in granulocytes already within the first month post-HSCT, and the intracellular virus copy numbers were similar in both indicated settings (Figures 4B,C).

In order to address the notion that granulocytes are not the only TTV reservoir in PB of immunocompetent children, erythrocyte and thrombocyte preparations were investigated for presence of the virus. Erythrocytes isolated from ten patients of the control cohort, seven of whom had tested TTV-positive in leukocyte preparations, tested negative for the virus. Moreover, platelet concentrates from 14 different healthy donors were analyzed for the presence of TTV, and the virus was detected in six instances (43%). The median virus copy number per platelet determined by RQ-PCR was 0.6 (range 0.2–7.5). To determine whether the TTV positivity found in platelet concentrates might be attributable to residual plasma present in the preparations, densely packed platelets derived by centrifugation of the concentrates were tested in parallel by RQ-PCR and revealed identical results in three of the six positive samples, suggesting that platelets can carry either internalized or adherent particles of the virus. The distribution and quantity of TTV in different PB cells are summarized in Table 2.

**Table 2:** Distribution and quantity of TTV in plasma and in leukocyte subsets.

Patients	Cell type/material	TTV positive	TTV copies/cell or ml^b^
HSCT patients^a^	Plasma	43/43 (100%)	7.46E+06 (0–1.85E+09)
	Granulocytes	19/19 (100%)	29.82 (0.1–3.87E+03)
Control cohort	Plasma	22/30 (73%)	5.43E+03 (0–3.12E+06)
	Granulocytes	10/30 (33%)	0.45 (0–7.9)
	T, B, NK cells. Monocytes	0/30 (0%)	0
	Erythrocytes	0/10 (0%)	0
	Platelets	6/14 (44%)	0.6 (0–7.5)

*^*a*^50 days post-HSCT; ^*b*^median (range)*.

## Discussion

Several studies suggested a correlation of increasing TTV levels in PB and plasma with the immune status of patients after transplantation, and indicated the possibility of exploiting post-transplant quantitative TTV analyses in SOT and HSCT recipients particularly as a surrogate marker for immune reconstitution (Focosi et al., 2010; Maggi et al., 2010; Gorzer et al., 2015; Albert et al., 2017; Gilles et al., 2017; Wohlfarth et al., 2018). However, data available to date show a rather unclear relationship between TTV and the immune system, thus rendering conclusions concerning the relevance of TTV monitoring difficult (Wohlfarth et al., 2018). Our data revealed no significant correlation between the lymphocyte counts and TTV levels at +30, +60, or +100 days post-transplant. The presence of constant or even slightly decreasing TTV levels during the first month post-transplant documented in the present study has also been described in a recent report (Albert et al., 2017), however, other observations indicated an increase of TTV immediately after transplantation (Gorzer et al., 2015). Data on the incidence and the potential impact of TTV expansion in pediatric transplant recipients have not been available, with the exception of a single report on high expression levels of various cytokines and the chemokine MCP-3 in association with TTV-positivity (Zanotta et al., 2015). Nevertheless, the putative role of the virus or the potential clinical use of its monitoring in the pediatric post-transplant setting remains enigmatic.

In agreement with earlier studies, our analysis of plasma specimens derived from HSCT recipients showed increasing TTV loads during the first 3 to 4 months post-transplant (Maggi et al., 2010; Gorzer et al., 2015; Wohlfarth et al., 2018), followed by gradually decreasing virus copy numbers in plasma to pre-transplantation levels over a period of about 2 years. The observed TTV kinetics in plasma might therefore reflect a correlation of the virus copy numbers with impairment of the immune system and its recovery post-transplant. It might be of relevance to consider that the variety of TTV genotypes increases in transplant recipients (Focosi et al., 2010) as a result of allografting and the common transfusion of blood products from various donors during the post-transplant course. Although it is unknown whether contemporaneous presence of multiple TTV genotypes in the circulation might have any impact on patients undergoing allogeneic HSCT, an effect on TTV replication during immunosuppression, particularly in patients without detectable TTV before transplantation, cannot be entirely excluded. Moreover, the absence of TTV in children below the age of 3 years might reflect the first contact and infection with the virus.

We observed elevated TTV copy numbers not only in plasma but also in stool specimens tested at the same time points, which might be attributable to the shedding of leukocytes carrying the virus into the intestines. This finding supported the possibility that TTV replication occurs in specific leukocyte subsets, and this notion was assessed by testing individual cell types isolated by flow-sorting at different time points after HSCT. Our data obtained in pediatric patients show that replication of the virus occurs in CD15^+^ cells representing neutrophilic granulocytes, the most abundant fraction of the granulocytes, and expansion of the virus generally starts 1 month after transplantation, upon engraftment of these cells. This finding might suggest that sufficiently high granulocyte numbers might be a prerequisite for efficient TTV expansion post-transplant, and could therefore provide a possible explanation for the delayed onset of rapid replication of the virus. Indeed, TTV copy numbers revealed a significant correlation with the number of neutrophils at +30 and +60 days after HSCT. A previous study reported a drop of TTV levels in patients treated with anti-thymocyte globulin (ATG) which was more pronounced than in patients treated with basiliximab, a weaker T lymphocyte inhibiting antibody. The possible effect of anti-T lymphocyte drugs on TTV levels provided the basis for the suggestion that T lymphocytes are the host cells for TTV replication (Focosi et al., 2015). This concept is not supported by our findings which did not reveal presence of the virus in any lymphocyte subset isolated from PB during the post-transplant course. In partial agreement with our observations, Takahashi et al. (2002) found the highest TTV levels in granulocytes derived from a small number of individuals and two log_10_ lower levels in other hematopoietic cells. Our findings indicated not only the presence of the virus in granulocytes but revealed a clear increase of TTV copy numbers in these cells with peak levels around 100 days post-transplant. It is necessary to consider that the life span of peripheral granulocytes is very short, generally within the range of a few days only, but our analyses of myeloid cells derived from the bone marrow also showed evidence for the virus. Although granulocytes are an important site of TTV replication, as demonstrated by the data presented, they may not serve as host cells for persistence of the virus. The exploitation of diverse cell types for persistence and replication of individual viruses is a well-known phenomenon. Viral persistence requires the infection of cells with a long life span, whereas replication, which is commonly associated with cell lysis, can occur in short-lived cell types providing the environment conducive for viral multiplication. In line with this notion, similar studies on human adenoviruses performed by us and others showed persistence of the virus in tonsillar and mucosal T lymphocytes (Garnett et al., 2002; Roy et al., 2011; Kosulin et al., 2013), whereas replication occurred in epithelial cells of the lungs or the intestine (Lam et al., 2015; Kosulin et al., 2016). Although we did not find TTV in T cells isolated by flow-sorting from PB, its persistence in these cells cannot be excluded because virus-infected T cells may not occur at sufficiently high frequencies in the circulation, and the intracellular virus concentrations may be very low.

The observation of rather constant TTV plasma levels during the first month post-transplant, the detection of rising plasma virus levels with documented replication in granulocytes during the ensuing 2 to 3 months, and the subsequent gradual return of the virus copy numbers to pre-transplant levels in the pediatric HSCT recipients investigated prompted us to compare the findings to a control cohort of immunocompetent children. The occurrence of TTV positivity in the plasma of patients prior to HSCT reflecting the state of persistence was very similar to the control group, revealing 79 and 73% of virus-positive individuals, respectively. However, TTV was identified in granulocytes in only one third of the children within the control cohort, while all HSCT recipients invariably tested positive for the virus in granulocytes by day +50 post-transplant. This implies that the virus is also carried by other cells in the immunocompetent control patients. However, in agreement with the findings in HSCT recipients, we could not detect the virus in isolated lymphocytes. In search for a carrier of TTV in PB, we identified presence of the virus in plasma-depleted platelet concentrates, raising the possibility that TTV may be taken up or adhere to thrombocytes. This notion is supported by the fact that several platelet receptors are known to bind viruses such as Epstein-Barr virus, hepatitis virus C, adenovirus, rotavirus, or human immunodeficiency virus (Chabert et al., 2015), but little information is available on the interactions between platelets and viruses. The drop of platelet counts associated with severe bleeding observed during some viral infections might be due to platelet destruction mediated by antibodies, by platelet-leukocyte aggregation or by direct viral effects on platelets. However, the assessment of possible effects mediated by the presence of TTV in or on platelets will require further investigation. Ongoing studies addressing the interactions of TTV with the human host will undoubtedly unveil the potential secrets of this currently enigmatic virus.

## Author Contributions

KK designed the study and carried out the experiments, analyzed and interpreted the data, and wrote the manuscript. SK carried out the experiments and analyzed the data. HP, AL, and VW provided patient samples and were involved in data collection. RG was involved in data analysis and interpretation. TL designed the study, was involved in data interpretation and manuscript preparation.

## Conflict of Interest Statement

The authors declare that the research was conducted in the absence of any commercial or financial relationships that could be construed as a potential conflict of interest.

## References

[B1] Albert E. Solano C. Pascual T. Torres I. Macera L. Focosi D. (2017). Dynamics of Torque Teno virus plasma DNAemia in allogeneic stem cell transplant recipients. *J. Clin. Virol.* 94 22–28. 10.1016/j.jcv.2017.07.001 28710997

[B2] Bendinelli M. Pistello M. Maggi F. Fornai C. Freer G. Vatteroni M. L. (2001). Molecular properties, biology, and clinical implications of TT virus, a recently identified widespread infectious agent of humans. *Clin. Microbiol. Rev.* 14 98–113. 10.1128/CMR.14.1.98-113.2001 11148004 PMC88963

[B3] Burra P. Masier A. Boldrin C. Calistri A. Andreoli E. Senzolo M. (2008). Torque Teno virus: any pathological role in liver transplanted patients? *Transplant. Int.* 21 972–979. 10.1111/j.1432-2277.2008.00714.x 18564988

[B4] Chabert A. Hamzeh-Cognasse H. Pozzetto B. Cognasse F. Schattner M. Gomez R. M. (2015). Human platelets and their capacity of binding viruses: meaning and challenges? *BMC Immunol.* 16:26. 10.1186/s12865-015-0092-1 25913718 PMC4411926

[B5] Chung J. Y. Han T. H. Koo J. W. Kim S. W. Seo J. K. Hwang E. S. (2007). Small anellovirus infections in Korean children. *Emerg. Infect. Dis.* 13 791–793. 10.3201/eid1305.061149 18044047 PMC2738455

[B6] De Vlaminck I. Khush K. K. Strehl C. Kohli B. Luikart H. Neff N. F. (2013). Temporal response of the human virome to immunosuppression and antiviral therapy. *Cell* 155 1178–1187. 10.1016/j.cell.2013.10.034 24267896 PMC4098717

[B7] Deng X. Terunuma H. Handema R. Sakamoto M. Kitamura T. Ito M. (2000). Higher prevalence and viral load of TT virus in saliva than in the corresponding serum: another possible transmission route and replication site of TT virus. *J. Med. Virol.* 62 531–537. 10.1002/1096-9071(200012)62:4<531::AID-JMV20>3.0.CO;2-C 11074484

[B8] Focosi D. Macera L. Boggi U. Nelli L. C. Maggi F. (2015). Short-term kinetics of torque teno virus viraemia after induction immunosuppression confirm T lymphocytes as the main replication-competent cells. *J. Gen. Virol.* 96 115–117. 10.1099/vir.0.070094-0 25304651

[B9] Focosi D. Maggi F. Albani M. Macera L. Ricci V. Gragnani S. (2010). Torquetenovirus viremia kinetics after autologous stem cell transplantation are predictable and may serve as a surrogate marker of functional immune reconstitution. *J. Clin. Virol.* 47 189–192. 10.1016/j.jcv.2009.11.027 20034850

[B10] Fritsch G. Witt V. Dubovsky J. Matthes S. Peters C. Buchinger P. (1999). Flow cytometric monitoring of hematopoietic reconstitution in myeloablated patients following allogeneic transplantation. *Cytotherapy* 1 295–309. 10.1080/0032472031000141265 20426555

[B11] Garnett C. T. Erdman D. Xu W. Gooding L. R. (2002). Prevalence and quantitation of species C adenovirus DNA in human mucosal lymphocytes. *J. Virol.* 76 10608–10616. 10.1128/JVI.76.21.10608-10616.2002 12368303 PMC136639

[B12] Gilles R. Herling M. Holtick U. Heger E. Awerkiew S. Fish I. (2017). Dynamics of Torque Teno virus viremia could predict risk of complications after allogeneic hematopoietic stem cell transplantation. *Med. Microbiol. Immunol.* 206 355–362. 10.1007/s00430-017-0511-4 28702856

[B13] Gorzer I. Jaksch P. Kundi M. Seitz T. Klepetko W. Puchhammer-Stockl E. (2015). Pre-transplant plasma Torque Teno virus load and increase dynamics after lung transplantation. *PLoS One* 10:e0122975. 10.1371/journal.pone.0122975 25894323 PMC4404260

[B14] Kosulin K. Geiger E. Vecsei A. Huber W. D. Rauch M. Brenner E. (2016). Persistence and reactivation of human adenoviruses in the gastrointestinal tract. *Clin. Microbiol. Infect.* 22 381.e1–381.e8. 10.1016/j.cmi.2015.12.013 26711435

[B15] Kosulin K. Hoffmann F. Clauditz T. S. Wilczak W. Dobner T. (2013). Presence of adenovirus species C in infiltrating lymphocytes of human sarcoma. *PLoS One* 8:e63646. 10.1371/journal.pone.0063646 23671688 PMC3646006

[B16] Lam E. Ramke M. Warnecke G. Schrepfer S. Kopfnagel V. Dobner T. (2015). Effective apical infection of differentiated human bronchial epithelial cells and induction of proinflammatory chemokines by the highly pneumotropic human adenovirus type 14p1. *PLoS One* 10:e0131201. 10.1371/journal.pone.0131201 26168049 PMC4500402

[B17] Maggi F. Bendinelli M. (2009). Immunobiology of the Torque teno viruses and other anelloviruses. *Curr. Top. Microbiol. Immunol.* 331 65–90. 10.1007/978-3-540-70972-5_5 19230558

[B18] Maggi F. Focosi D. Albani M. Lanini L. Vatteroni M. L. Petrini M. (2010). Role of hematopoietic cells in the maintenance of chronic human torquetenovirus plasma viremia. *J. Virol.* 84 6891–6893. 10.1128/JVI.00273-10 20410268 PMC2903282

[B19] Maggi F. Fornai C. Zaccaro L. Morrica A. Vatteroni M. L. Isola P. (2001). TT virus (TTV) loads associated with different peripheral blood cell types and evidence for TTV replication in activated mononuclear cells. *J. Med. Virol.* 64 190–194. 10.1002/jmv.1035 11360252

[B20] Maggi F. Pifferi M. Fornai C. Andreoli E. Tempestini E. Vatteroni M. (2003). TT virus in the nasal secretions of children with acute respiratory diseases: relations to viremia and disease severity. *J. Virol.* 77 2418–2425. 10.1128/JVI.77.4.2418-2425.2003 12551979 PMC141071

[B21] Masouridi-Levrat S. Pradier A. Simonetta F. Kaiser L. Chalandon Y. Roosnek E. (2016). Torque teno virus in patients undergoing allogeneic hematopoietic stem cell transplantation for hematological malignancies. *Bone Marrow Transplant.* 51 440–442. 10.1038/bmt.2015.262 26551776

[B22] Nishizawa T. Okamoto H. Konishi K. Yoshizawa H. Miyakawa Y. Mayumi M. (1997). A novel DNA virus (TTV) associated with elevated transaminase levels in posttransfusion hepatitis of unknown etiology. *Biochem. Biophys. Res. Commun.* 241 92–97. 10.1006/bbrc.1997.7765 9405239

[B23] Okamoto H. (2009). History of discoveries and pathogenicity of TT viruses. *Curr. Top. Microbiol. Immunol.* 331 1–20. 10.1007/978-3-540-70972-5_1 19230554

[B24] Okamoto H. Akahane Y. Ukita M. Fukuda M. Tsuda F. Miyakawa Y. (1998). Fecal excretion of a nonenveloped DNA virus (TTV) associated with posttransfusion non-A-G hepatitis. *J. Med. Virol.* 56 128–132. 10.1002/(SICI)1096-9071(199810)56:2<128::AID-JMV5>3.0.CO;2-A 9746068

[B25] Roy S. Calcedo R. Medina-Jaszek A. Keough M. Peng H. Wilson J. M. (2011). Adenoviruses in lymphocytes of the human gastro-intestinal tract. *PLoS One* 6:e24859. 10.1371/journal.pone.0024859 21980361 PMC3184098

[B26] Schiemann M. Puchhammer-Stockl E. Eskandary F. Kohlbeck P. Rasoul-Rockenschaub S. Heilos A. (2017). Torque Teno virus load-inverse association with antibody-mediated rejection after kidney transplantation. *Transplantation* 101 360–367. 10.1097/TP.0000000000001455 27525643 PMC5268087

[B27] Spandole S. Cimponeriu D. Berca L. M. Mihaescu G. (2015). Human anelloviruses: an update of molecular, epidemiological and clinical aspects. *Arch. Virol.* 160 893–908. 10.1007/s00705-015-2363-9 25680568

[B28] Takahashi M. Asabe S. Gotanda Y. Kishimoto J. Tsuda F. Okamoto H. (2002). TT virus is distributed in various leukocyte subpopulations at distinct levels, with the highest viral load in granulocytes. *Biochem. Biophys. Res. Commun.* 290 242–248. 10.1006/bbrc.2001.6183 11779160

[B29] Watzinger F. Suda M. Preuner S. Baumgartinger R. Ebner K. Baskova L. (2004). Real-time quantitative PCR assays for detection and monitoring of pathogenic human viruses in immunosuppressed pediatric patients. *J. Clin. Microbiol.* 42 5189–5198. 10.1128/JCM.42.11.5189-5198.2004 15528714 PMC525141

[B30] Wohlfarth P. Leiner M. Schoergenhofer C. Hopfinger G. Goerzer I. Puchhammer-Stoeckl E. (2018). Torquetenovirus dynamics and immune marker properties in patients following allogeneic hematopoietic stem cell transplantation: a prospective longitudinal study. *Biol. Blood Marrow Transplant.* 24 194–199. 10.1016/j.bbmt.2017.09.020 29032273

[B31] Zanotta N. Maximova N. Campisciano G. Del Savio R. Pizzol A. Casalicchio G. (2015). Up-regulation of the monocyte chemotactic protein-3 in sera from bone marrow transplanted children with torquetenovirus infection. *J. Clin. Virol.* 63 6–11. 10.1016/j.jcv.2014.11.028 25600596

